# Occurrence and Molecular Characteristics of Extended-Spectrum Beta-Lactamase-Producing Enterobacterales Recovered From Chicken, Chicken Meat, and Human Infections in Sao Paulo State, Brazil

**DOI:** 10.3389/fmicb.2021.628738

**Published:** 2021-06-22

**Authors:** Marita Vedovelli Cardozo, Apostolos Liakopoulos, Michael Brouwer, Arie Kant, Lucas José Luduvério Pizauro, Mariana Monezi Borzi, Dik Mevius, Fernando Antonio de Ávila

**Affiliations:** ^1^Department of Veterinary Pathology, Faculty of Agricultural and Veterinary Sciences, São Paulo State University (Unesp), Jaboticabal, Brazil; ^2^Department of Bacteriology and Epidemiology, Wageningen Bioveterinary Research, Wageningen University and Research, Lelystad, Netherlands; ^3^Department of Tecnology, Faculty of Agricultural and Veterinary Sciences, São Paulo State University (Unesp), Jaboticabal, Brazil

**Keywords:** antibiotic resistance, plasmids, extended spectrum beta lactamases (ESBLs), poultry, food chain

## Abstract

This study aimed to investigate the phylogenetic diversity and epidemiology of extended-spectrum beta-lactamase (ESBL)-producing *Escherichia coli* and *Klebsiella pneumoniae* from chicken, chicken meat, and human clinical isolates in Sao Paolo, Brazil, and characterize their respective ESBL-encoding plasmids. Three hundred samples from chicken cloaca, chicken meat, and clinical isolates were phenotypically and genotypically assessed for ESBL resistance. Isolates were identified by MALDI TOF-MS and further characterized by MLST analysis and phylogenetic grouping. ESBL genes were characterized and their location was determined by I-Ceu-I-PFGE and Southern blot, conjugation, transformation, and PCR-based replicon typing experiments. Thirty-seven ESBL-producing isolates (28 *E. coli* and 9 *K. pneumoniae*) that were positive for the *bla*_*CTX–M*–1_ or *bla*_*CTX–M*–2_ gene groups were obtained. Two isolates were negative in the transformation assay, and the chromosomal location of the genes was deduced by Southern blot. The *bla*_*CTX–M*_ genes identified were carried on plasmid replicon-types X1, HI2, N, FII-variants, I1 and R. The *E. coli* isolates belonged to nine sequence types, while the *K. pneumoniae* isolates belonged to four sequence types. The *E. coli* isolates belonged to phylotype classification groups A, B1, D, and F. This study demonstrated that isolates from cloacal swabs, chicken meat, and human feces had genetic diversity, with a high frequency of *bla*_*CTX–M*–15_ among chickens, chicken meat, and human feces. Thus, this reinforces the hypothesis that chickens, as well as their by-products, could be an important source of transmission for ESBL-producing pathogens to humans in South America.

## Introduction

Enterobacterales carrying extended-spectrum β-lactamase (ESBLs) genes with resistance to third- and fourth-generation cephalosporins have been detected widely in livestock (Wu et al., 2018). The role of chicken meat as a potential source of multidrug-resistant bacteria that carries ESBL genes have been demonstrated in several countries including China (Wu et al., 2018), Canada (Ghosh et al., 2019), The Netherlands (Liakopoulos et al., 2016), Senegal (Vounba et al., 2019), and Brazil (Casella et al., 2017).

Brazil draws attention as the world’s largest chicken meat and derivatives exporter (USDA, 2019). Therefore, European countries have demonstrated concerns regarding Brazilian imported chicken meat due to its possible role in transferring antibiotic-resistant strains from food to humans (Liakopoulos et al., 2016). Although it has been shown that human-to-human transmission in the open community has a greater impact on transmission of ESBL-producing isolates than other putative sources (Mughini-Gras et al., 2019), this concern originates from the fact that genetic determinants, such as plasmids, encoding CTX-M enzymes may be transmitted to humans *via* the food chain (Cyoia et al., 2019) and/or direct contact with animals or the environment (Mughini-Gras et al., 2019).

Several studies in Brazil have shown the presence of ESBL-encoding isolates in animals, chicken meat, and humans, but none of them has demonstrated the presence and the relationship of CTX-M-producing Enterobacteriaceae in the food chain, comprising of broilers, chicken meat, and the consumers. Therefore, we aimed to investigate the origin, phylogenetic diversity, and epidemiology of ESBL-producing *Escherichia coli* and *Klebsiella pneumoniae* isolates from chickens and chicken meat, and their relationship with those causing clinical symptoms in humans in Sao Paulo, Brazil, as well as characterize their respective ESBL-encoding genes and plasmid replicon types.

## Materials and Methods

### Bacterial Sampling, Identification, and Phenotypic Characterization

A total of 300 samples, from the northwest region of the Sao Paulo, from two farms, two clinics and two slaughterhouses within a 80 km radius, were obtained between February and October of 2014 in Sao Paulo, Brazil, consisting of: (1) 100 cloacal swabs of clinically healthy chickens originated from two different farms in the same state [50 from Farm 1 (F1) and 50 from Farm 2 (F2)], (2) 100 chicken meat samples at a local retail (1 g each) [50 from Supermarket 1 (S1), and 50 from Supermarket 2 (S2)], and (3) 100 samples of human feces collected from an equal number of patients with gastrointestinal disease without prior antibiotic treatment of two different local hospitals [50 from Hospital 1 (H1), and 50 from Hospital 2 (H2)]. After collection, samples were transferred to a selective pre-enrichment broth (Luria–Bertani broth supplemented with 1 mg/L cefotaxime) and incubated overnight at 37°C. Subsequently, they were cultured on selective MacConkey agar plates supplemented with 1 mg/L cefotaxime (Sigma-Aldrich, Germany), and incubated for 24 h at 37°C. Thereafter, five morphologically different colonies per sample were tested for ESBL production using a combination disk test as previously described (Liakopoulos et al., 2016). The species of the recovered ESBL-producing isolates was determined by MALDI-TOF mass spectrometry (MALDI Biotyper, Bruker, Germany).

### ESBL-Gene Typing

The genomic DNA was extracted by DNeasy Blood and Tissue Kit (QIAGEN, Germany). The presence of ESBL genes was assessed by microarray analysis using the Check-MDR CT-101 (Check-Points, Wageningen, Netherlands) and characterized by polymerase chain reaction (PCR) and sequence analysis as previously described (Dierikx et al., 2013). Sequence data were analyzed using Sequencher version 4.2 (Gene Codes Corporation, United States), and the sequences obtained were compared to ones deposited in GenBank.

### Bacterial and Plasmid Typing

All *E. coli* and *K. pneumoniae* isolates were characterized by multilocus sequence typing (MLST) according to Achtman’s^1^ and Pasteur’s^2^ schemes, respectively. *E. coli* phylotyping was performed according to Clermont et al. (2013). After transformation and/or conjugation, plasmid characterization was performed by PCR-based replicon typing (PBRT) on transformants and/or transconjugants as previously described (Carattoli et al., 2005).

### Genetic Support of the *bla*_*CTX–M*_ Genes

The localization of ESBL genes on plasmids was assessed by transformation and/or conjugation experiments. For transformation experiments, the plasmids were extracted using Qiagen Plasmid Midi Kits (Qiagen, Netherlands) and electro-transformed in ElectroMaxTM H10BTM cells (Gibco Invitrogen, United States). Conjugation assays were performed in Luria–Bertani medium (LB-medium) using a rifampicin-resistant, indole-negative *E. coli* K12 strain as the recipient (Liakopoulos et al., 2016). Transformants were selected on MacConkey agar containing 1 mg/L cefotaxime, whereas transconjugants on MacConkey agar contained 1 mg/L cefotaxime and 100 mg/L rifampicin. The chromosomal location of the ESBL genes, when necessary, was confirmed by I-Ceu-I-PFGE followed by Southern blot hybridization, as previously described (Liakopoulos et al., 2016).

## Results and Discussion

**FIGURE 1:**
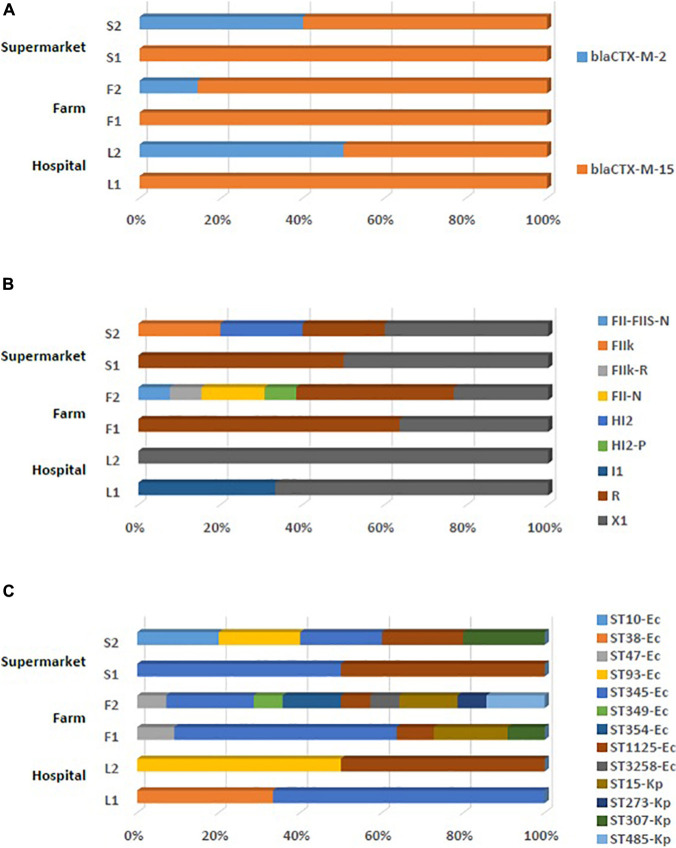
Distribution of ESBL gene types **(A)**, plasmid replicon types **(B)**, and isolate sequence types **(C)** among the ESBL-producing isolates recovered from each of the reservoirs tested. Refer to Table 1 for the more detailed molecular characteristics of each isolate recovered.

From the MacConkey agar screening coupled with the combination disk test, we recovered 25 ESBL-producing isolates from 100 chicken cloacal samples (25%), seven isolates from 100 chicken meat samples (7%), and five isolates from 100 human fecal samples (5%). MALDI-TOF MS revealed that these 37 isolates composed of nine *K. pneumoniae* and 28 *E. coli* strains. Interestingly, ESBL-producing *K. pneumoniae* is so far detected rarely among poultry (Zhuo et al., 2013; Daehre et al., 2018; Projahn et al., 2019). A micro-array showed that our isolates were positive for *bla*_*CTX–M*–1_ or *bla*_*CTX–M*–2_ group genes. Through sequencing, we have shown that all *bla*_*CTX–M*–1_-group-harboring isolates encoded the *bla*_*CTX–M*–15_ gene, and all *bla*_*CTX–M*–2_-group encoded the *bla*_*CTX–M*–2_ gene, with *bla*_*CTX–M*–15_ being the most prevalent ESBL gene (86.4%; 32/37) among isolates of all sources (Figure 1A). Specifically, our data indicated that the *bla*_*CTX–M*–15_ gene was present in 91.3% (23/25) of the chicken cloacal isolates, in 71.4% (5/7) of the chicken meat isolates, and in 83.3% (4/5) of the human feces isolates (Table 1). Despite the *bla*_*CTX–M*–2_ and *bla*_*CTX–M*–8_ genes being the most predominant ESBL genes so far in South America, the *bla*_*CTX–M*–15_ gene has recently emerged in clinical isolates and is now detected as often as the *bla*_*CTX–M*–2_ gene in humans, while it has only been reported sporadically from chickens in Brazil (Botelho et al., 2015; Ferreira et al., 2016; Casella et al., 2018). Either these cases reflect direct contamination through human handling or the potential emergence of the *bla*_*CTX–M*–15_ gene in chickens on farms, they pose the risk for further spread of the *bla*_*CTX–M*–15_ gene within the poultry production pyramid. We identified only five isolates encoding the *bla*_*CTX–M*–2_ gene**,** in particularly two *E. coli* from cloaca, two from chicken meat and one from human feces, contrary to the previous studies documenting the high prevalence of the *bla*_*CTX–M*–2_ gene among isolates recovered from chicken meat in Brazil (Casella et al., 2018). Overall, the detection of *E. coli* and *K. pneumoniae* isolates carrying *bla*_*CTX–M*_ genes raises concerns about the broad dissemination of these antimicrobial resistance determinants in Brazil.

**TABLE 1:** Isolate origin, identification, ESBL gene, plasmid type, sequence type, and phylotype.

Isolate	Origin	Reservoir	MALDI-TOF MS	PCR-ESBL	Sequencing	Plasmid	MLST	Phylogroup
1	Cloacal swab	F1	*E. coli*	CTX-M-1g	*bla*_*CTX–M*–15_	IncX1	47	B1
2	Cloacal swab	F1	*E. coli*	CTX-M-1g	*bla*_*CTX–M*–15_	IncX1	345	B1
3	Cloacal swab	F1	*E. coli*	CTX-M-1g	*bla*_*CTX–M*–15_	IncX1	1125	B1
4	Cloacal swab	F1	*E. coli*	CTX-M-1g	*bla*_*CTX–M*–15_	R	345	B1
5	Cloacal swab	F1	*E. coli*	CTX-M-1g	*bla*_*CTX–M*–15_	R	345	B1
6	Cloacal swab	F1	*E. coli*	CTX-M-1g	*bla*_*CTX–M*–15_	R	345	B1
7	Cloacal swab	F1	*E. coli*	CTX-M-1g	*bla*_*CTX–M*–15_	R	345	B1
8	Cloacal swab	F1	*E. coli*	CTX-M-1g	*bla*_*CTX–M*–15_	R	345	B1
9	Cloacal swab	F1	*K. pneumonie*	CTX-M-1g	*bla*_*CTX–M*–15_	IncX1	307	-
10	Cloacal swab	F1	*K. pneumonie*	CTX-M-1g	*bla*_*CTX–M*–15_	R	15	-
11	Cloacal swab	F1	*K. pneumonie*	CTX-M-1g	*bla*_*CTX–M*–15_	R	15	-
12	Cloacal swab	F2	*E. coli*	CTX-M-1g	*bla*_*CTX–M*–15_	IncN IncFII	354	F
13	Cloacal swab	F2	*E. coli*	CTX-M-1g	*bla*_*CTX–M*–15_	IncN IncFII	354	F
14	Cloacal swab	F2	*E. coli*	CTX-M-1g	*bla*_*CTX–M*–15_	IncN IncFII IncFIIS	349	D
15	Cloacal swab	F2	*E. coli*	CTX-M-1g	*bla*_*CTX–M*–15_	IncX1	345	B1
16	Cloacal swab	F2	*E. coli*	CTX-M-1g	*bla*_*CTX–M*–15_	IncX1	345	B1
17	Cloacal swab	F2	*E. coli*	CTX-M-1g	*bla*_*CTX–M*–15_	IncX1	1125	B1
18	Cloacal swab	F2	*E. coli*	CTX-M-1g	*bla*_*CTX–M*–15_	R	345	D
20	Cloacal swab	F2	*E. coli*	CTX-M-2g	*bla*_*CTX–M*–2_	IncHI2 IncP	47	A
19	Cloacal swab	F2	*E. coli*	CTX-M-2g	*bla*_*CTX–M*–2_	N/A	3258	D
21	Cloacal swab	F2	*K. pneumonie*	CTX-M-1g	*bla*_*CTX–M*–15_	R	15	-
22	Cloacal swab	F2	*K. pneumonie*	CTX-M-1g	*bla*_*CTX–M*–15_	R	15	-
23	Cloacal swab	F2	*K. pneumonie*	CTX-M-1g	*bla*_*CTX–M*–15_	R	485	-
24	Cloacal swab	F2	*K. pneumonie*	CTX-M-1g	*bla*_*CTX–M*–15_	R	485	-
25	Cloacal swab	F2	*K. pneumonie*	CTX-M-1g	*bla*_*CTX–M*–15_	R IncFIIK	273	-
27	Human feces	H1	*E. coli*	CTX-M-1g	*bla*_*CTX–M*–15_	IncX1	345	B1
28	Human feces	H1	*E. coli*	CTX-M-1g	*bla*_*CTX–M*–15_	IncX1	345	B1
26	Human feces	H1	*E. coli*	CTX-M-1g	*bla*_*CTX–M*–15_	IncI1	38	D
29	Human feces	H2	*E. coli*	CTX-M-1g	*bla*_*CTX–M*–15_	IncX1	1125	A
30	Human feces	H2	*E. coli*	CTX-M-2g	*bla*_*CTX–M*–2_	N/A	93	B1
31	Chicken meat	S1	*E. coli*	CTX-M-1g	*bla*_*CTX–M*–15_	IncX1	1125	D
32	Chicken meat	S1	*E. coli*	CTX-M-1g	*bla*_*CTX–M*–15_	R	345	B1
33	Chicken meat	S2	*E. coli*	CTX-M-1g	*bla*_*CTX–M*–15_	IncX1	345	B1
34	Chicken meat	S2	*E. coli*	CTX-M-1g	*bla*_*CTX–M*–15_	IncX1	1125	B1
35	Chicken meat	S2	*E. coli*	CTX-M-2g	*bla*_*CTX–M*–2_	IncHI2	10	Non-typable
36	Chicken meat	S2	*E. coli*	CTX-M-2g	*bla*_*CTX–M*–2_	R	93	D
37	Chicken meat	S2	*K. pneumonie*	CTX-M-1g	*bla*_*CTX–M*–15_	IncFIIK	307	-

*N/A: Not applied as the ESBL gene for these isolates was confirmed to be encoded on the chromosome.
*

Transformation experiments revealed that the ESBL genes were plasmid-encoded in 35 of the 37 isolates with these plasmids belonging to diverse replicon-types (Figure 1B). In particular, plasmid replicon-types detected for the *bla*_*CTX–M*–15_ gene were R, X1, FIIK, I1, N-FII(-FIIs), and R-FIIk, whereas for the *bla*_*CTX–M*–2_ gene were R and HI2(-P). In the two *E. coli* isolates that were negative for transformation and the subsequent conjugation experiments, suggesting that the *bla*_*CTX–M*–2_ gene was not located on a plasmid (Table 1), we confirmed the chromosomal location of the *bla*_*CTX–M*–2_ gene by I-Ceu-I-PFGE followed by Southern blot hybridization (Supplementary Figure 1). Although the *bla*_*CTX–M*–2_ gene has been previously documented in different genetic backgrounds as well as on plasmids highlighting multiple integration events and transmission pathways, it has been mostly reported to be chromosomally encoded (Casella et al., 2018). In our study, only a limited number of *bla*_*CTX–M*–2_ genes were chromosomally encoded, suggesting that plasmids are important facilitators of their spread among the recovered isolates. Overall, our data highlight the contribution of plasmids on the epidemiology of ESBL-producing Enterobacterales of poultry and human origin in Sao Paulo, Brazil.

Clermont’s classification demonstrated that our *E. coli* isolates belonged to A, B1, D, and F groups with one isolate being not classifiable. The B1 was the most prevalent with 64.7% (11/28) of cloacal isolates belonging to this group, followed by 17.6% (3/17) of D, 11.7% (2/17) of F, and 5.8% (1/17) of A. Similarly, B1 was the most prevalent group among the isolates that we recovered from human clinical samples (60%; 3/5) and chicken meat (50%; 3/6). Isolates assigned to the B1 group have been previously associated with mostly intestinal pathogenic *E. coli* with high virulent potential in animal models (Morcatti et al., 2018).

Multilocus sequence typing classification demonstrated that the 28 *E. coli* isolates belonged to nine sequence types (ST10, ST38, ST47, ST93, ST345, ST349, ST354, ST1125, and ST3258) with the ST345 and ST1125 being the most prevalent ones (46.4 and 17.8%, respectively) (Figure 1C). *K. pneumoniae* isolates were assigned to four sequence types (ST15, ST273, ST307, and ST485) with ST15 being the most prevalent (44.4%) one (Figure 1C). Of note, we observed some known epidemic clones among the *E. coli* (i.e., ST10 and ST38) and *K. pneumoniae* (i.e., ST15 and ST307) isolates (Woodford et al., 2011; Mathers et al., 2015; Navon-Venezia et al., 2017). *K. pneumoniae* ST15 isolates harboring *bla*_*CTX–M*–15_, are emerging among patients with respiratory tract infections in China (An et al., 2012; Xu et al., 2019) and have been previously isolated from companion animals in Paris (Morcatti et al., 2018). As previously described, our data reveal clonal diversity among the recovered isolates and highlight Brazilian poultry meat as a reservoir of ExPEC lineages (i.e., ST10) (Casella et al., 2018).

As observed by Casella et al. (2018), we show genetic identity in the ESBL gene, plasmid type, isolate ST, and phylogroup suggesting clonal similarity in *K. pneumoniae* isolates between F1 and F2 (*bla*_*CTX–M*–15_, R, ST15) but also *E. coli* isolates among (1) F1, F2, H1, and S2 (*bla*_*CTX–M*–15_, IncX1, ST345/B1); (2) F1 and S1 (*bla*_*CTX–M*–15_, R, ST345/B1); and (3) F1, F2, and S2 (*bla*_*CTX–M*–15_, IncX1, ST1125/B1) (Table 1). In addition, we observed genetic identity in ESBL gene and plasmid type suggesting plasmid spread among (1) F1, F2, H1, H2, S1, and S2 (*bla*_*CTX–M*–15_, IncX1) and (2) F1, F2, and S1 (*bla*_*CTX–M*–15_, R) (Table 1). Overall, our data highlight a complex epidemiology of ESBL-producing Enterobacterales driven by both clones and plasmids, as well as the potential transmission of these clones and plasmids along the poultry meat chain to humans and/or vice versa.

In conclusion, we demonstrated that despite their overall genetic diversity, isolates from cloacal swabs, chicken meat, and human feces present genetic similarities highlighting that Brazilian chickens, as well as their by-products, may be an important source of transmission for ESBL-producing pathogens to humans. In addition, we indicated the occurrence and high frequency of *E. coli* and *K. pneumoniae* isolates harboring the *bla*_*CTX–M*–15_ gene from chicken and chicken meat products in South America for the first time.

## Data Availability Statement

The original contributions presented in the study are included in the article/Supplementary Material, further inquiries can be directed to the corresponding author/s.

## Author Contributions

MC conceptualized and designed the study. AL and LP aided with data analysis, and manuscript preparation and revision. AK aided in data acquisition. MBo in data acquisition and manuscript preparation. All authors read, contributed to, and approved the final manuscript.

## Conflict of Interest

The authors declare that the research was conducted in the absence of any commercial or financial relationships that could be construed as a potential conflict of interest.

## Footnotes

1 http://mlst.ucc.ie/mlst/dbs/Ecoli

2 http://bigsdb.pasteur.fr/klebsiella/

## Supplementary Material

Supplementary Figure 1 Chromosomal localization of the *bla*_*CTX–M*–2_ gene by I-Ceu-I-PFGE and Southern blot hybridization. Columns 1 (Isolate 19) and 2 (Isolate 30) depict the hybridization results using an intragenic *bla*_*CTX–M*–2_ gene probe, whereas columns 3 (*S. enterica* ser. Braenderup strain H9812—marker), 4 (Isolate 19), and 5 (Isolate 30) depict the hybridization results using intragenic 16S rDNA probes.
