# Supplementary figures and images for: Occurrence and Molecular Characteristics of Extended-Spectrum Beta-Lactamase-Producing Enterobacterales Recovered From Chicken, Chicken Meat, and Human Infections in Sao Paulo State, Brazil
Source: Front Microbiol. 2021 Jun 22;12:628738. doi: 10.3389/fmicb.2021.628738 (PMC8259509; doi:10.3389/fmicb.2021.628738)

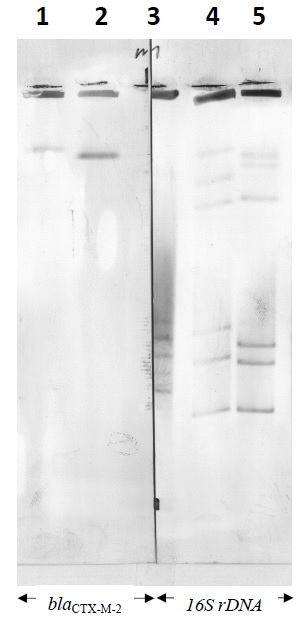

Supplement: Supplementary Figure 1 — Chromosomal localization of the blaCTX–M–2 gene by I-Ceu-I-PFGE and Southern blot hybridization. Columns 1 (Isolate 19) and 2 (Isolate 30) depict the hybridization results using an intragenic blaCTX–M–2 gene probe, whereas columns 3 (S. enterica ser. Braenderup strain H9812—marker), 4 (Isolate 19), and 5 (Isolate 30) depict the hybridization results using intragenic 16S rDNA probes. [file Image_1.jpeg]
